# Value of Nerve Biopsy in Patients With Latent Malignant Hemopathy and Peripheral Neuropathy: A Case Series

**DOI:** 10.1097/MD.0000000000000394

**Published:** 2015-01-26

**Authors:** Mathilde Duchesne, Stéphane Mathis, Philippe Corcia, Laurence Richard, Karima Ghorab, Arnaud Jaccard, Laurent Magy, Jean-Michel Vallat

**Affiliations:** From the Department of Pathology (MD); Department of Neurology, CHU Poitiers, University of Poitiers, Poitiers (SM); Department of Neurology, CHU Tours, Tours (PC); Department of Hematology, CHU Limoges (AJ); and Department of Neurology and Centre de Référence “Neuropathies périphériques rares”, CHU Limoges, Limoges, France (JMV).

## Abstract

Hematological malignancies include several diseases that may affect the peripheral nervous system (PNS) through various mechanisms. A common and challenging situation is represented by the occurrence of an active peripheral neuropathy in a patient with a supposed inactive hematological disorder.

We report clinical, electrophysiological, biological, and pathological data of 8 patients with latent malignant hemopathies (most were considered in remission): B-cell chronic lymphocytic leukemia in 3 patients, B-cell lymphoma in 1 patient, low-grade non-Hodgkin's lymphoma in 1 patient, Waldenström's macroglobulinemia in 1 patient, smoldering multiple myeloma in 1 patient, and monoclonal gammopathy of undetermined significance in 1 patient.

In all these cases, the nerve biopsy (NB) helped to diagnose the hematological relapse or detect a pathological mechanism linked to the hematological disorder: epineurial lymphocytic infiltration in 5 patients (including one with antimyelin-associated glycoprotein antibodies), cryoglobulin deposits in 1 patient, chronic inflammatory demyelinating polyneuropathy in 1 patient, and necrotizing vasculitis in 1 patient. In each case, pathological findings were crucial to select the adequate treatment, leading to an improvement in the neurological and biological manifestations.

These observations illustrate the value of NB and the need for active collaboration between neurologists and hematologists in such cases.

## INTRODUCTION

Peripheral neuropathy is recognized as a potential complication of numerous hematological malignancies^1^; it may also be secondary to their treatment: chemotherapy^2^ and radiation.^3^ Diagnosis and management of peripheral neurological complications of these diseases may be difficult, particularly when the neoplasm is thought inactive based on clinical and biological criteria. To illustrate this situation, we report 8 patients who all presented an active peripheral neuropathy associated with a latent malignant hemopathy. In most of these patients, the hematological disorder was considered to have responded to treatment and to be in remission. A direct or indirect link to hemopathies was envisaged, but could only be definitely proved by a nerve biopsy (NB), which was determinant in the management of these patients.

## MATERIALS AND METHODS

### Selection of Patients

Among patients who underwent NB in our neurology department in the past 10 years, there were 8 with active neuropathy as a complication of various latent hematological malignancies. The neuropathy could not be attributed to the side effects of chemotherapy. Patients underwent a detailed neurological examination and clinimetric evaluation: Overall Neuropathy Limitations Scale (ONLS)^4^ and Medical Research Council score (MRC).^5^ The clinical context and laboratory investigations ruled out other possible causes of neuropathy. Informed consents were obtained from all subjects for the NB, but this retrospective study does not require an ethics committee’ approval according to the current laws in our Hospital.

### Electrodiagnostic Studies

They were performed according to standard techniques.^6^

### Nerve Biopsy

After informed consent, NB was performed in all patients (sural or radial nerve) and processed as described elsewhere.^7^ One fragment was fixed in 10% formaldehyde then embedded in paraffin; another fragment was frozen for immunocytochemical analysis (anti-CD45, CD20, CD4, CD8, lambda light chain, kappa light chain, CD68). Congo red staining was performed systematically. One other fragment was fixed in 2.5% glutaraldehyde and embedded in Epon. Semi-thin sections were examined by light microscopy and ultrathin sections were examined using an electron microscope (EM); a few other fragments were embedded in London Resin White (LRW) for an immuno-EM study in case of a monoclonal gammopathy. In this case, direct immunofluorescence was carried out on frozen sections. Samples of the nerves were also teased.

## RESULTS

Results are presented in Tables 1 and 2. The details for each patient are the following:

**TABLE 1:**
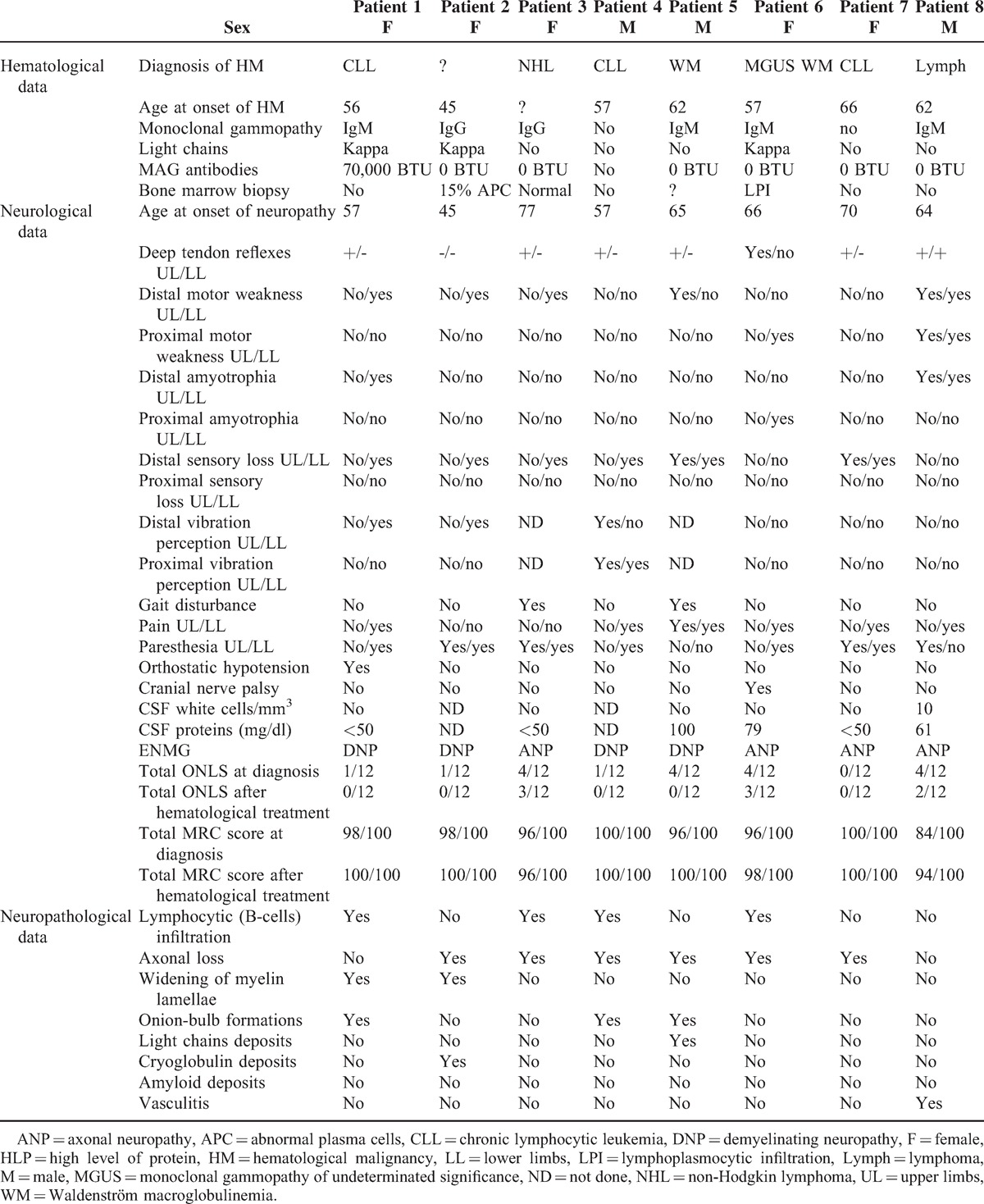
Summary of the Main Clinical, Pathological and Biological Findings of the Patients

**TABLE 2:**
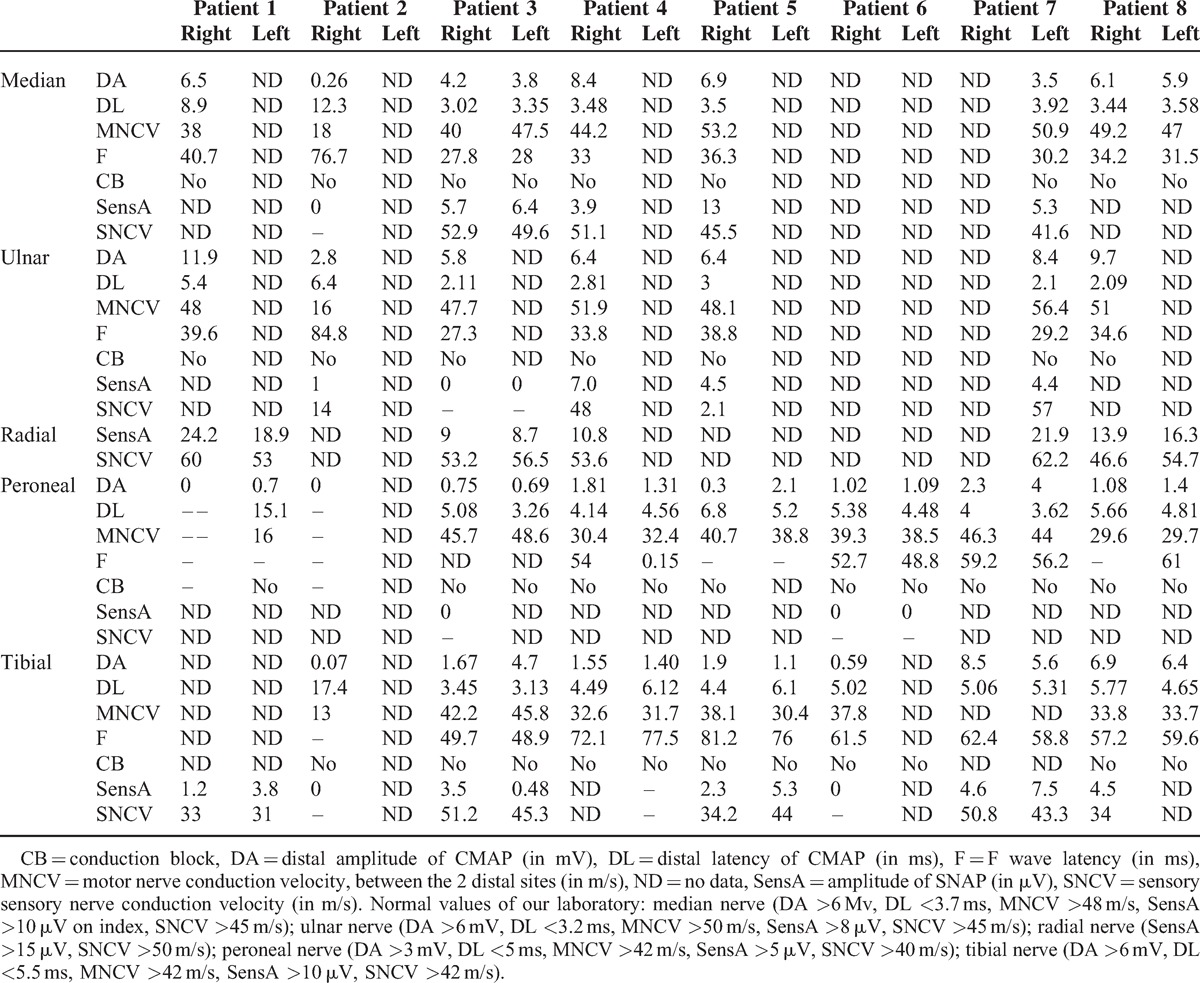
Electrophysiological Findings of Patients

### Patient 1

This 57-year-old woman had an incidental finding of asymptomatic B-cell chronic lymphocytic leukemia (B-CLL) with monoclonal gammopathy (IgM-Kappa); no treatment was required for the hemopathy. At that time, she suffered from mild paresthesia, with a sensation of cold feet. One year later, she presented with a slight distal symmetrical amyotrophy and weakness of the lower limbs (extension of the toes: 4/5 on MRC), absent Achilles deep tendon reflexes, and decrease of vibration perception in the feet (ONLS score: 1/12). NCV studies showed a sensorimotor, distal, symmetrical demyelinating polyneuropathy (Table 2). Blood cell count and examination revealed leukocytosis (16,400 cells/mm^3^; N < 10,000–12,530 cells/mm^3^ small lymphocytes, N < 4000). Other ancillary tests confirmed the IgM monoclonal gammopathy (7.3 g/L; N < 1.7) with a Kappa light chain (52.5 mg/L; N < 19.4) and a high level of antimyelin-associated glycoprotein (MAG) antibodies (>70,000 Bühlmann Titer Unit). Cerebrospinal fluid (CSF) was normal.

#### Pathology

**FIGURE 1:**
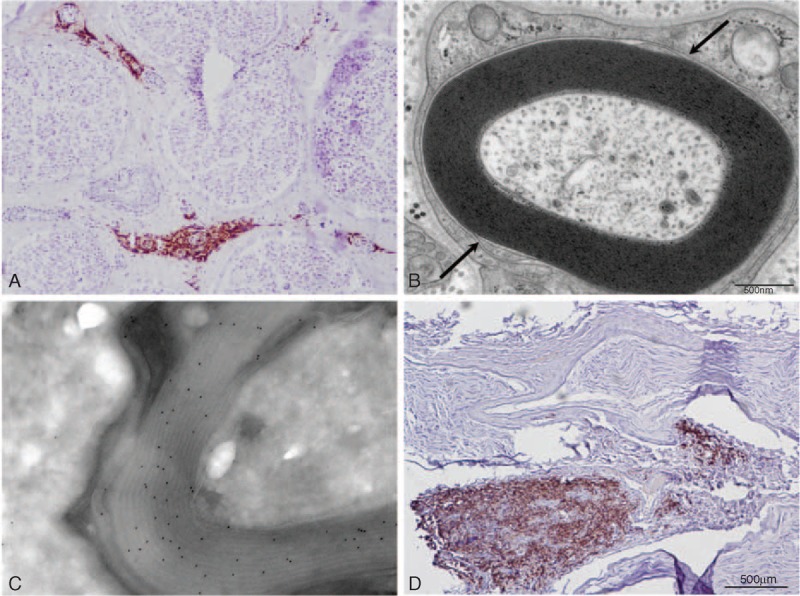
(A) Frozen transverse section of the sural nerve of patient 1 stained with anti-CD20 antibody. Several vessels in the epineurium are surrounded by B-cell infiltrates. (B) Electron micrograph of tranverse section of the sural nerve of patient 1 showing the typical widening of the most external myelin lamellae. (C) Immunoelectron micrograph of patient 2. Anti-kappa light chain immunogold staining shows that the monoclonal protein of the patient has infiltrated the myelin sheath. (D) Frozen transverse section of the sural nerve of patient 3 stained with anti-CD20 antibody. There is a massive infiltrate of B-cells in the epineurium.

A massive lymphocytic infiltration (CD20+ B-cells, with a few CD45+ T-cells and a few CD68+ macrophages) was detected in the epineurium (Figure 1A). Multiple polymerase chain reaction examination of RNA extracted from a frozen nerve sample confirmed the monoclonality of the B-cell infiltrate.^8^ Congo red-stained sections were normal. On semi-thin sections through EM, we observed chronic demyelinating lesions and characteristic widening of myelin lamellae (WML) as described in anti-MAG neuropathies (Figure 1B).

#### Outcome

Rituximab, fludarabine, and cyclophosphamide were prescribed, leading to an improvement: decrease in paresthesiae, but with persistent distal sensory loss in the lower limbs): After treatment, ONLS: 0/12 (MRC sum score in the four limbs: 100/100).

### Patient 2

The case of this 45-year-old woman was already published.^9^ She had a history of breast adenocarcinoma and complained of progressive distal paresthesia of the 4 limbs since a few months and 2 years later, presented with a distal weakness of the lower limbs (MRC:4/5). Generalized areflexia, symmetric distal loss of pin-prick and temperature of the lower limbs, and mild decrease of vibration perception in the feet were observed (ONLS: 1/12). The electrophysiological study showed a demyelinating sensorimotor polyneuropathy (Table 2). Ancillary tests demonstrated a high level of monoclonal IgG cryoglobulin (Kappa light chain). A bone marrow biopsy showed 15% infiltration by plasma cells with an abnormal appearance. The skeletal survey showed no osteolytic lesion and the final diagnosis was a smoldering multiple myeloma associated with a chronic inflammatory demyelinating polyradiculoneuropathy (CIDP). Plasma exchanges improved the biological abnormalities, but no neurological improvement was observed nor after treatment with steroids and intravenous immunoglobulin (IVIg). An NB was performed.

#### Pathology

A severe loss of myelinated fibers was observed on semi-thin sections. By EM, WML was frequent, and they were regularly spaced (33.9 ± 1.4 versus 15.2 ± 1.9 nm for spacing in normal myelin). Outside Schwann cells, small deposits of a granular substance were detected; immuno-EM demonstrated that they consisted of Kappa light chains (IgG), as well as those located inside the WML (Figure 1C).

#### Outcome

The patient was treated by an allogenic bone marrow graft and cyclophosphamide; neurological manifestations improved (ONLS: 0/12; MRC sum score: 100/100). Five years later, clinical examination was normal with no further monoclonal gammopathy.

### Patient 3

This 77-year-old woman, in remission of a low-grade non-Hodgkin lymphoma, was in therapeutic abstention since 10 years. She was referred to us because of recent symmetrical tingling in the hands and feet. Clinical examination showed gait ataxia, severe distal symmetrical hypoesthesia of the lower limbs, absent deep tendon reflexes in the lower limbs, and distal motor weakness of the lower limbs (flexion and extension of the feet was graded 4/5 on MRC: the total MRC score was 96/100; ONLS was 4/12). Nerve conduction study showed an axonal sensorimotor polyneuropathy mainly affecting the lower limbs (Table 2). Ancillary tests showed a hypothyroidism. Anti-ganglioside and anti-MAG antibodies were negative. No abnormality was observed on the bone marrow biopsy. CSF, bone marrow aspiration, salivary gland biopsy, and whole-body Positron Emission Tomography (PET) scan were normal.

#### Pathology

On a sural NB, multiple clusters of epineurial lymphocytic infiltrates (massive infiltration of CD20+ B-cells, with a few CD45+ T-cells) and some CD68+ macrophages (also in the endoneurial space) were observed (Figure 1D). Clonal B-cells were detected by conventional techniques (see above). Congo red-stained sections were normal. Semi-thin sections showed a severe axonal loss with a few ovoids indicating subacute or acute axonal lesions.

#### Outcome

Rituximab and fludarabine were started 1 year after the onset of the polyneuropathy, which remained relatively stable with moderate regression of sensory disturbances, whereas walking and gait were still affected. ONLS decreased (3/12), but the total MRC score was stable.

### Patient 4

This 63-year-old male patient had a history of B-CLL (CD5+, CD23+, FMC7+, F79+, and CD38+, with Kappa light chains) diagnosed 4 years earlier and in therapeutic abstention (grade A). The medical history comprised tuberculosis (total recovery) and a restless legs syndrome. He was admitted to the neurology department for paresthesia, pain, and hypoesthesia of the lower limbs. Hemopathy was still biologically stable without any tumoral syndrome, but the patient presented with weight loss. Clinical examination showed a hypoesthesia of the external part of the left leg and foot, but also a hyperesthesia of the left calf and of both soles of the feet (ONLS = 1/12; MRC sum score = 100/100). Achilles deep tendon reflexes were absent. Ancillary tests confirmed the presence in the serum of a B-cell clone of CD5+ and CD20+ (with Kappa light chains). Serum protein electrophoresis, Lyme serology, QuantiFERON, and anti-MAG antibodies were negative. Nerve conduction studies showed a demyelinating sensorimotor polyneuropathy of the lower limbs, with axonal loss (Table 2). A sural NB was finally performed.

#### Pathology

**FIGURE 2:**
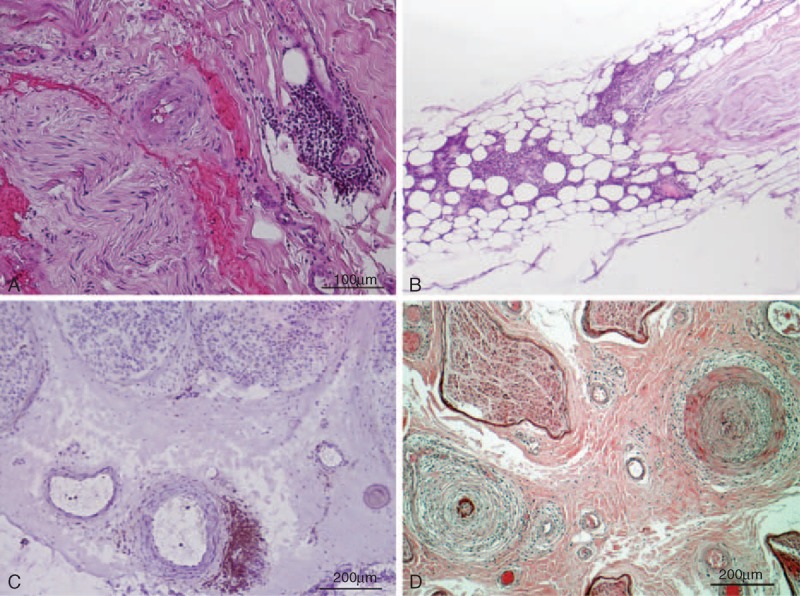
(A) Paraffin-embedded transverse section of the sural nerve of patient 4 stained with hematxylin-eosin, showing perivascular infiltrate of mononuclear cells in the epineurium. (B) Paraffin-embedded longitudinal section of the radial nerve of patient 5, showing massive infiltrates of mononuclear cells in the epineurium. (C) Frozen tranverse section of the sural nerve of patient 7 stained with anti-CD45 antibody, showing a perivascular epineurial T-cell infiltrate. (D) Paraffin-embedded transverse section of the sural nerve of patient 8, stained with hematoxylin-eosin. Two middle-size vessels of the epineurium display the typical aspect of necrotizing vasculitis.

We observed small B-cell infiltrates (Figure 2A). Semi-thin and ultra-thin sections showed a severe loss of large myelinated fibers and demyelinating-remyelinating lesions.

#### Outcome

Six courses of monthly association of rituximab, fludarabine, and cyclophosphamide were done. The course was marked by an improvement in the general condition of the patient, and a regression of the sensory signs and symptoms (ONLS: 0/12; MRC: 100/100). The patient's state remained stable thereafter.

### Patient 5

This 65-year-old man had been treated during 1 year for Waldenström macroglobulinemia (total IgM >80 g/L; N = 0.5–1.5) at the age of 62 (with cyclophosphamide, fludarabine, and rituximab). This treatment was stopped in view of a clinical and biological remission. Two years later, he complained of gait disturbance and pain in the toes. Four years after the end of the chemotherapy, he developed “burning” sensations in the hands, with mild weakness on prehension and spreading of the fingers. There was gait disturbance, anesthesia of the feet, hypoesthesia of both hands, hypopallesthesia of both feet and hands, and absent Achilles deep tendon reflexes (other reflexes were weak). ONLS: 4/12, MRC sum score: 96/100. The electrophysiological study demonstrated a demyelinating sensorimotor polyneuropathy (with a severe decrease of SNAP of the upper limb). Laboratory examination confirmed a high level of IgM (48 g/L). Anti-ganglioside and anti-MAG antibodies were negative. CSF examination showed an increase of protein (1.07 g/L; N = 0.15–0.5) and 12 white cells per mm^3^ (no clonal cells). Because of worsening of the symptoms, a radial NB was performed.

#### Pathology

An epineurial and endoneurial infiltration by numerous foci of Kappa light chain-positive B-lymphocytes was detected (Figure 2B). Clonal B-cells were detected. Semi-thin sections showed a severe axonal loss with few ovoids relative to subacute or acute unspecific axonal lesions.

#### Outcome

A dramatic neurological improvement (normalization of the clinical examination) was observed after a treatment with 6 courses of R-mini Cyclophosphamide + Hydroxydaunorubicin + Onconvin (= vincristine) + Prednisone(CHOP) chemotherapy (ONLS: 0/12, MRC: 100/100).

### Patient 6

This 66-year-old woman complained of pain, tingling, paresthesia, and weakness of the lower limbs for a few weeks. She had a history of IgM Kappa monoclonal gammopathy of undeterminated significance (MGUS) diagnosed 9 years earlier. She complained of asthenia; there was no lymph node or spleen enlargement. The neurological examination found symmetric weakness and amyotrophy of the quadriceps muscles (MRC: 2/5), absent deep tendon reflexes in the lower limbs, diplopia caused by a paresis of the left abducens nerve, and no major sensory disturbance (ONLS: 4/12; MRC sum score: 96/100). The spinal cord MRI was normal, and the electrophysiological study found an asymmetric axonal sensorimotor neuropathy affecting the four limbs (Table 2). CSF demonstrated a slightly elevated protein level (0.79 g/L; N = 0.15–0.5) without cells. Ancillary tests revealed: IgM-Kappa was 40.6 g/L (N = 0.53–2.09 g/L); clonal B-cells were observed in blood (CD5−, CD23+, FMC7+, CD79b−, CD20+, lambda monotypic). The bone marrow aspiration confirmed a significant plasma cell and lymphocytic proliferation (30%) compatible with the progression of MGUS to Waldenström macroglobulinemia.

#### Pathology

A massive mononuclear cell infiltration (CD20+ B-cells mainly, and a few CD45+ T-cells and CD68+ macrophages) was detected in the epineurium. Clonal cells were detected using conventional technique. Congo red-stained sections were normal. Semi-thin sections revealed a slight axonal loss with some ovoids.

#### Outcome

The patient was treated with rituximab, bendamustine, and methotrexate, in association with intrathecal administration of cytarabine and methylpredinsolone. After 6 courses, there was an overall increase in motor and disability scores (MRC sum score = 98/100, ONLS = 3/12).

### Patient 7

This 70-year-old woman had a history of B-CLL diagnosed 4 years ago (Matutes score was 4). She was treated with cyclophosphamide for 1 year, before 12 courses of the association cyclophosphamide-fludarabine in view of recurrence of the disease. At that time, she was considered to be in remission. One year later, she complained of tingling and sensory loss in the right upper limb and the anterior part of the left leg, then the right foot (6 months later). She also presented pain in her left lower limb. No motor weakness (MRC = 100/100) and no signs of autonomic dysfunction were observed. Deep tendon reflexes were absent in the lower limbs (ONLS: 0/12). There was no lymph node or spleen enlargement. Nerve conduction studies showed an axonal and predominantly sensory polyneuropathy (Table 2). Ancillary tests, CSF, and spinal MRI were normal. Because of the history of B-CLL, a sural NB was performed.

#### Pathology

Several mononuclear cell infiltrates were observed around small vessels diffusely distributed in the nerve parenchyma. Immunochemistry revealed only T lymphocytes with few macrophages (Figure 2C). Semi-thin and ultrathin examinations showed an axonal loss associated with a significant number of demyelinating and remyelinating lesions. The diagnosis of CIDP was retained.

#### Outcome

After 3 courses of IVIg (every 6 weeks), pain and sensory symptoms improved markedly (ONLS: 0/12, MRC: 100/100).

### Patient 8

This 63-year-old man was admitted to the department of Neurology for neck and back pain, associated with asymmetrical paresthesiae and pain in the lower limbs (predominantly on the left side). At that time, he was considered in remission of a B-cell lymphoma of ileum and cecum localizations, having been treated 9 years earlier by 4 courses of Cyclophosphamide + Hydroxydaunorubicin + Onconvin (= vincristine) + Prednisone. The electrophysiological study was in favor of a sensorimotor axonal asymmetrical neuropathy (Table 2). Otherwise, in his medical history, he had herpes zoster, Basedow disease, and myocardial infarction. Neurological examination showed distal amyotrophy and overall global mild weakness of the 4 limbs (total MRC was 84/100), with weak deep tendon reflexes (ONLS: 4/12). Laboratory investigations revealed a recurrence of a low-grade lymphoma (CD79a+ and CD20−) without monoclonal gammopathy in the serum. The whole-body [18F]-2-Fluoro-2-Deoxy-D-Glucose PET scan showed metabolic activity in numerous lymph nodes (tonsils, neck, groins, axilla, and mediastinum); a biopsy of a retroxiphisternal ganglion indicated a relapse of the lymphoma. The association of idarubicine, cyclophosphamide, and dexamethasone induced improvement in the biological features (but with pain persisting in the lower limbs). CSF showed 10 white cells per mm^3^ with a mild elevated protein level (61 mg/dL). Except for significant positivity of the anti-Sjögren's-Syndrome-related antigen A antibodies (ratio = 2.6, positive if >1.4), immunological analysis was normal. Because of the persisting pain, a sural NB was performed.

#### Pathology

Lesions of necrotizing vasculitis (with infiltration by numerous mononuclear cells and fibrinoid necrosis of the vessel walls of middle-sized epineurial arterioles) were observed (Figure 2D). These cells were neither B-cells nor eosinophils; most of them were T lymphocytes and macrophages. No clonal B-cells were detected by conventional techniques.

#### Outcome

A steroid treatment (1 mg/kg/d during several months) was prescribed, with a significant neurological improvement (MRC: 94/100) and a dramatic decrease in pain (ONLS: 2/12) after 6 months.

## DISCUSSION

A variety of mechanisms may explain the association between malignant hemopathy and neuropathy: infiltration of the nerve parenchyma by malignant cells, endoneurial deposits of immunoglobulin, infiltration of the immunoglobulin between myelin lamellae, POEMS (polyneuropathy, organomegaly, endocrinopathy, M-protein, skin changes) syndrome, deposits of amyloid, or autoimmune process such as CIDP.^10^ In the context of a hematologic disorder, some patients may develop a neuropathy, which is directly or indirectly linked to the malignant hemopathy, although this disease is either considered latent or in remission after a specific treatment. Coordination between hematologists and neurologists is very useful to determine whether a specific treatment should be begun or if the ongoing treatment needs to be modified. All the cases presented here benefitted from this collaboration. Clearly, the treatment depends on the mechanism(s) of nerve lesions, which in our cases could only be determined by NB using different techniques to establish a link or not between the hemopathy and the polyneuropathy. Ultrastructural examination (in combination with routine histological and immunopathological examinations) was decisive as in our patients 1 and 2.

A possible mechanism is the presence of deposits of immunoglobulin in the endoneurial space that can be detected by light microscopy if they are big enough. They were very small in our patient 2, so that they would not have been detected without an EM study and a specific confirmation by immuno-EM. The first symptoms of nerve involvement began 2 years before the first clinical examination, and at that time a monoclonal IgG Kappa was noted; a diagnosis of smouldering multiple myeloma was envisaged, so it was decided to defer chemotherapy. It took more than four years to prove that the polyneuropathy was induced by immunoglobulin endoneurial deposits. The mechanisms of destruction of nerve fibers by immunoglobulin deposits is unclear and such deposits can be observed in all types of monoclonal dysglobulinemia (IgG, IgA, or IgM).^10^ Amyloid deposits need to be differentiated from immunoglobulin deposits with specific Congo red staining. Sometimes, the monoclonal dysglobulinemia exhibits properties of cryoglobulin, as in our patient 2; immuno-EM study has clearly indicated that the endoneurial deposits and the circulating cryoglobulin were identical.^10-12^

Another possible mechanism of neuropathy in the context of a latent malignant hemopathy is the infiltration of nerve parenchyma by malignant cells (neurolymphomatosis),^13^ which is mostly due to B-cell lymphomas (as our cases 1, 3, 4, 5, and 6), and rarely secondary to T-cell or natural killer cell lymphomas.^14,15^ The incidence of neurolymphomatosis is estimated to be 3% in patients with newly diagnosed NHL.^16^ However, only 20% of patients with neurolymphomatosis are known to have systemic lymphoma at diagnosis of the nerve involvement;^14^ such infiltrations can be only diagnosed by an NB when CSF examination does not allow confirming the presence of clonal B-cells. Clinical manifestations are various: involvement of cranial nerves, painful neuropathy, pure sensory or sensory-motor signs, and asymmetrical involvement of several nerves (multiplex mononeuropathy) or only of one nerve (mononeuropathy).^14^ Rarely patients present with a symmetrical neuropathy as in our patients 1, 3, 5, and 6,^13^ sometimes mimicking a CIDP^1^ or a Guillain–Barré syndrome.^17^ The most common neurological localization of lymphoma is meningoradicular, which explains the abnormal CSF in 97% of cases.^18^ However, if the neuropathy reveals a malignant lymphoma in 26% of cases (NB leading to a diagnosis in 88% of cases), CSF analysis is positive in only 40% of the total cases of lymphoma;^15^ in most of our cases, CSF was completely normal.

CIDP is probably underestimated; its prevalence is estimated between 1 and 7.7 per 100,000 people in different studies.^19^ CIDP may be associated with various disorders as hematological disorders such as lymphoma or MGUS, with no clear mechanism for such concurrent diseases.^19^ The possibility of misdiagnosing neurolymphomatosis as isolated CIDP is high.^1^ Conversely, it is also possible to misdiagnose a CIDP in a context of neuropathy with lymphoma; the major difference between intraneural lymphomatous infiltration and CIDP is that T-lymphocytes and only a few B-lymphocytes are usually observed in CIDP NB.^20^ This was the case of our patient 7 who had a B-lymphoma and a CIDP phenotype; an NB showed T-lymphocyte infiltrates and macrophages and she improved with IVIg. In such cases, if there are enough cells in the nerve parenchyma, detection of clonal cells may also be helpful using specific techniques.^8^

In such a context, a pitfall may be the concomitant occurrence of two different diseases. In our patient 8, NB revealed that the polyneuropathy was induced by a necrotizing vasculitis involving only peripheral nerves, and which had no direct relationship with the lymphoma. In such a case, an NB is mandatory for the diagnosis and to rule out other etiologies. Necrotizing vasculitis might be considered as a rare paraneoplastic syndrome in the context of a malignant hemopathy or of various types of carcinoma.^21^ However, intraneural lymphoproliferative infiltration may rarely mimic polyarteritis nodosa.^22^

Finally, 2 coexisting mechanisms may explain the nerve lesions, but can only be detected by careful studies of an NB. In our patient 1, an abnormal proliferation of malignant B-cells was associated with WML induced by an anti-MAG IGM, which has also been described associated with intraneural amyloid deposits.^23^

Nowadays, cytostatic drugs and recombinant monoclonal antibodies can produce molecular remission of hematological malignancies. However, our observations show that such a remission is not systematically a synonym of total remission of the hematologic disease; this apparent remission may sometimes hide neurological complications such as NP.

In malignant hematologic disorders, the incidence of specific intranervous lesions is underestimated, as NB is not available in many centers. The indications of an NB to explore an NP in this context should be systematically discussed, as a treatment adapted to the nerve lesions can be prescribed.

## References

[1] Tomita M Koike H Kawagashira Y Clinicopathological features of neuropathy associated with lymphoma. *Brain* 2013; 136:2563–2578. 23884813 10.1093/brain/awt193

[2] Windebank AJ Grisold W Chemotherapy-induced neuropathy. *J Peripher Nerv Syst* 2008; 13:27–46. 18346229 10.1111/j.1529-8027.2008.00156.x

[3] Mathis S Dumas P Neau JP La neuropathie motrice pure, une complication rare de la radiotherapie: trois observations et une revue de la litterature. *Rev Med Intern* 2007; 28:377–387. 10.1016/j.revmed.2007.01.025 17337314

[4] Graham RC Hughes RA A modified peripheral neuropathy scale: the Overall Neuropathy Limitations Scale. *J Neurol Neurosurg Psychiatry* 2006; 77:973–976. 16574730 10.1136/jnnp.2005.081547 PMC2077620

[5] Tally N O’Connor S Clinical examination: a systematic guide to physical diagnosis. 5th ed Sidney: Churchill Linvingstone; 2006.

[6] Kimura J Dyck P Thomas P Nerve conduction studies and needle electromyography. *Peripheral neuropathy* 4th ed Philadelphia: Elsevier Saunders; 2005 899–970.

[7] Vallat JM Vital A Magy L An update on nerve biopsy. *J Neuropathol Exp Neurol* 2009; 68:833–844. 19606069 10.1097/NEN.0b013e3181af2b9c

[8] van Dongen JJ Langerak AW Bruggemann M Design and standardization of PCR primers and protocols for detection of clonal immunoglobulin and T-cell receptor gene recombinations in suspect lymphoproliferations: report of the BIOMED-2 Concerted Action BMH4-CT98-3936. *Leukemia* 2003; 17:2257–2317. 14671650 10.1038/sj.leu.2403202

[9] Vallat JM Magy L Sindou P IgG neuropathy: an immunoelectron microscopic study. *J Neuropathol Exp Neurol* 2005; 64:386–390. 15892295 10.1093/jnen/64.5.386

[10] Vallat JM Magy L Richard L Contribution of electron microscopy to the study of neuropathies associated with an IgG monoclonal paraproteinemia. *Micron* 2008; 39:61–70. 17291771 10.1016/j.micron.2006.12.005

[11] Vallat JM Magy L Richard L Intranervous immunoglobulin deposits: an underestimated mechanism of neuropathy. *Muscle Nerve* 2008; 38:904–911. 18563720 10.1002/mus.21057

[12] Vallat JM Desproges-Gotteron R Leboutet MJ Cryoglobulinemic neuropathy: a pathological study. *Ann Neurol* 1980; 8:179–185. 7425571 10.1002/ana.410080208

[13] Grisold W Briani C Vass A Malignant cell infiltration in the peripheral nervous system. *Handb Clin Neurol* 2013; 115:685–712. 23931810 10.1016/B978-0-444-52902-2.00040-0

[14] Baehring JM Damek D Martin EC Neurolymphomatosis. *Neuro Oncol* 2003; 5:104–115. 12672282 10.1215/S1522-8517-02-00017-0 PMC1920674

[15] Grisariu S Avni B Batchelor TT Neurolymphomatosis: an International Primary CNS Lymphoma Collaborative Group report. *Blood* 2010; 115:5005–5011. 20368468 10.1182/blood-2009-12-258210 PMC3710441

[16] Gan HK Azad A Cher L Neurolymphomatosis: diagnosis, management, and outcomes in patients treated with rituximab. *Neuro Oncol* 2010; 12:212–215. 20150388 10.1093/neuonc/nop021 PMC2940573

[17] Jiang QL Pytel P Rowin J Disseminated intravascular large-cell lymphoma with initial presentation mimicking Guillain-Barre syndrome. *Muscle Nerve* 2010; 42:133–136. 20544922 10.1002/mus.21648

[18] Vallat JM De Mascarel HA Bordessoule D Non-Hodgkin malignant lymphomas and peripheral neuropathies-13 cases. *Brain* 1995; 118:1233–1245. 7496783 10.1093/brain/118.5.1233

[19] Eldar AH Chapman J Guillain Barre syndrome and other immune mediated neuropathies: diagnosis and classification. *Autoimmun Rev* 2014; 13:525–530. 24434363 10.1016/j.autrev.2014.01.033

[20] Ben-Smith A Gaston JS Barber PC Winer JB Isolation and characterisation of T lymphocytes from sural nerve biopsies in patients with Guillain-Barre syndrome and chronic inflammatory demyelinating polyneuropathy. *J Neurol Neurosurg Psychiatry* 1996; 61:362–368. 8890774 10.1136/jnnp.61.4.362 PMC486576

[21] Hutterer M Steurer M Hoftberger R Polyarteritis nodosa complicating multiple myeloma - a case report and review of the literature. *Clin Neuropathol* 2014; 33:143–151. 24220008 10.5414/NP300686

[22] Mouthon L Guilpain P Martin A Lymphoplasmacytic lymphoma associated with polyradiculoneuritis and cryoglobulinemia mimicking polyarteritis nodosa. *Presse Med* 2007; 36:623–626. 17287105 10.1016/j.lpm.2006.11.021

[23] Julien J Vital C Vallat JM IgM demyelinative neuropathy with amyloidosis and biclonal gammopathy. *Ann Neurol* 1984; 15:395–399. 6430211 10.1002/ana.410150415

